# Diverse roles of actin in *C. elegans *early embryogenesis

**DOI:** 10.1186/1471-213X-7-142

**Published:** 2007-12-24

**Authors:** Nathalie Velarde, Kristin C Gunsalus, Fabio Piano

**Affiliations:** 1 New York University, Department of Biology and Center for Comparative Functional Genomics, 100 Washington Square East, New York, NY 10003, USA

## Abstract

**Background:**

The actin cytoskeleton plays critical roles in early development in *Caenorhabditis elegans*. To further understand the complex roles of actin in early embryogenesis we use RNAi and *in vivo *imaging of filamentous actin (F-actin) dynamics.

**Results:**

Using RNAi, we found processes that are differentially sensitive to levels of actin during early embryogenesis. Mild actin depletion shows defects in cortical ruffling, pseudocleavage, and establishment of polarity, while more severe depletion shows defects in polar body extrusion, cytokinesis, chromosome segregation, and eventually, egg production. These defects indicate that actin is required for proper oocyte development, fertilization, and a wide range of important events during early embryogenesis, including proper chromosome segregation. *In vivo *visualization of the cortical actin cytoskeleton shows dynamics that parallel but are distinct from the previously described myosin dynamics. Two distinct types of actin organization are observed at the cortex. During asymmetric polarization to the anterior, or the establishment phase (Phase I), actin forms a meshwork of microfilaments and focal accumulations throughout the cortex, while during the anterior maintenance phase (Phase II) it undergoes a morphological transition to asymmetrically localized puncta. The proper asymmetric redistribution is dependent on the PAR proteins, while both asymmetric redistribution and morphological transitions are dependent upon PFN-1 and NMY-2. Just before cytokinesis, actin disappears from most of the cortex and is only found around the presumptive cytokinetic furrow. Finally, we describe dynamic actin-enriched comets in the early embryo.

**Conclusion:**

During early *C. elegans *embryogenesis actin plays more roles and its organization is more dynamic than previously described. Morphological transitions of F-actin, from meshwork to puncta, as well as asymmetric redistribution, are regulated by the PAR proteins. Results from this study indicate new insights into the cellular and developmental roles of the actin cytoskeleton.

## Background

Studying the actin cytoskeleton in a developmental context has been challenging due to its ubiquitous distribution and multi-faceted functions. The actin cytoskeleton is assembled from actin monomers ("globular", or G-actin) that polymerize into rigid fibers ("filamentous", or F-actin), also called microfilaments. Actin filament dynamics and architecture are regulated by a large number of actin-binding proteins, leading to a variety of higher-order actin-based structures that provide structural support, enable motility, and help organize the contents of the cell [1,2]. Actin is involved in such diverse cellular processes as cytokinesis, segregation of cell-fate determinants, motility and cell migration, and organelle transport [1]. Actin also plays key roles in fertilization and establishment of polarity [3,4], as well as endocytosis and movement of intracellular pathogens [5].

In *C. elegans*, an intact actin cytoskeleton is important for polarization of the one-cell embryo, which in turn is essential for proper developmental patterning (reviewed in [6-8]). The anteroposterior (AP) axis of the embryo is established shortly after fertilization in response to a cue from the sperm, which enters at or near the future posterior pole [9] and brings the centrosome/microtubule organizing center (MTOC). Sperm entry also triggers the completion of oocyte meiosis I and II, swirling of zygote cytoplasm, and the secretion of a chitinous eggshell. Once fertilized, the one-cell embryo becomes polarized and divides asymmetrically. This requires a group of "PAR" (*par*titioning-defective) proteins (see [6] for review), which become cortically localized to the anterior (PAR-3, PAR-6, and PKC-3) and posterior (PAR-2 and PAR-1) halves of the embryo in distinct establishment and maintenance phases) [10], defining anterior and posterior domains at the one-cell stage.

The first indications that the actin cytoskeleton is involved in establishing or maintaining asymmetry in the one-cell embryo were based on observations that disruption of microfilaments using Latrunculin A and cytochalasin D leads to loss of polarity [11,12]. Other actin-binding proteins such as *nmy-2, mlc-4, pod-1*, and *pfn-1 *also disrupt polarity [13-16]. Loss-of-function and conditional genetic mutations and gene-specific RNAi have shown that three of the five actin genes in *C. elegans *(*act-1, act-2*, and *act-3*) play a redundant role in the embryo [4,11,12]. Conditional dominant *act-2 *alleles cause abnormal and striking membrane ingressions and protrusions (but no cytokinesis or polarity defects). In contrast, incomplete depletion of *act-1 *by RNAi, which also affects other actins ([17], see Additional File 1: Supplementary Figure 1), results in a failure to extrude polar bodies, loss of normal spindle displacement, and cytokinesis failure; further depletion leads to cessation of egg production [18].

Localization studies using fixed preparations showed that actin filaments [19,20] and an actin-binding protein, POD-1 [21,22], become asymmetrically localized in a similar manner to the anterior PAR proteins. *In vivo *imaging of green fluorescent protein (GFP) fused to non-muscle myosin II heavy chain (NMY-2::GFP) subsequently revealed that the early embryo contains a dynamic network of contractile myosin fibers that appears to become destabilized near the point of sperm entry, leading to a cortical flow of NMY-2 and anterior PAR proteins toward the anterior pole [23]. Consistent with this observation, the centrosome appears to locally inhibit RHO-1, a small GTPase that regulates the actin cytoskeleton, by posterior cortical exclusion of its guanine exchange factor (RhoGEF), ECT-2 [24]. Furthermore, paternally provided CYK-4, a Rho-GTPase activating protein (RhoGAP), mediates a localized decrease in RHO-1 activity at the posterior [25]. The combined effect of these activities triggers anterior cortical flow and the cascade of events leading to cortical polarity. The PAR proteins reciprocally influence cortical actomyosin dynamics, suggesting that polarization involves interplay between these two systems [23,24]; however, a detailed study of F-actin dynamics during polarity establishment has yet to be described.

The actin cytoskeleton also plays a significant role in several types of intracellular transport. In both yeast and vertebrate cells, actin helps both to recruit proteins necessary to direct endocytic vesicle internalization and trafficking and to generate forces that power vesicle movement [5,26-30]. Several kinds of bacterial and viral intracellular pathogens co-opt the host cell's actin cytoskeleton to assemble an actin-rich "comet tail" at one pole (reviewed in [5,31-33]), and similar comet tails have been observed in association with endocytic vesicles [26-28,30]. *In vivo *studies and assays in reconstituted motility systems have shown that propulsion of actin comets involves the Arp2/3-mediated nucleation of actin filaments on the surface of the parasite or organelle, followed by extension and cross-linking to form a tail that propels the cargo through the cytoplasm using the force generated by actin assembly at the surface (see [5,31-33] for review). The *C. elegans *one-cell embryo appears to undergo active endocytosis, based on rapid labeling of the plasma membrane and punctate intracellular structures with the lipophilic dye FM 4–64 in laser-permeabilized embryos [22]. Actin is reported to play a role in the normal distribution of both early endosomes [34] and endoplasmic reticulum (ER) [35] in the first cell cycle. However, so far no direct role for actin in vesicle internalization and no actin-based vesicle propulsion have been described in this system.

To study actin-dependent processes during early embryogenesis and uncover potential differences in their sensitivity to actin levels, we used RNAi to deplete all three actin isoforms expressed in the early embryo and analyzed the progression of increasingly severe loss of function across a time course. Using GFP::MOE, a fusion protein that decorates actin filaments with GFP and recapitulates the distribution of F-actin *in vivo*, we provide a detailed characterization of the dynamic behavior of F-actin at the cortex throughout early embryogenesis. F-actin gradually becomes localized to the anterior of the one-cell embryo and displays a striking transition in morphology from a fibrous meshwork to a pattern of punctate foci upon pronuclear meeting. Key regulators of polarity and of the actin cytoskeleton are required for normal F-actin dynamics at the cortex. PAR-6 activity is required for the redistribution of F-actin to the anterior, whereas the posterior PAR proteins and CDC-42 are needed to maintain this asymmetric distribution and establish a precise boundary. Depletion of PFN-1, NMY-2, or ARX-1 severely disrupts the morphology of the cortical actin network. We also describe for the first time the presence of highly motile actin comets in *C. elegans *embryos.

## Results

### Actin is required for specific processes during early embryogenesis

**Figure 1:**
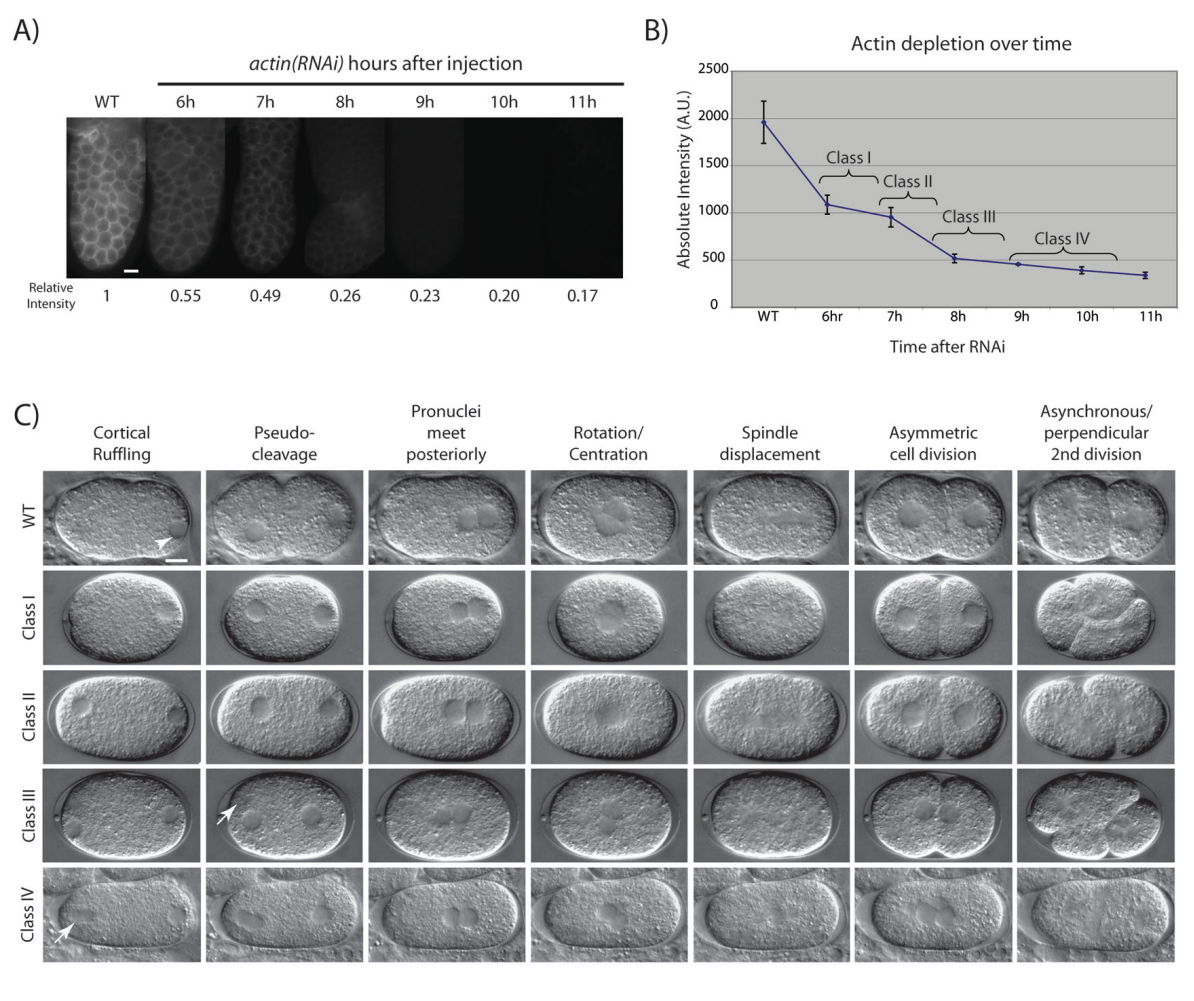
**Progressive depletion of actin over time in *C. elegans *gonads and embryos**. (A) Fixed gonads stained with anti-actin antibody in wild-type and *actin(RNAi) *treated animals at successive timepoints after injection of dsRNA, visualized with FITC-conjugated secondary antibody. The distal end of the gonad is shown. For each timepoint, the maximum signal intensity was quantified in each of three sampled areas per gonad, and the average of these was then compared to WT to obtain a measure of relative intensity. Scale bar is 10 μm. (B) Graph of actin depletion over time in *actin(RNAi) *gonads based on data shown in (A). Brackets indicate approximate time intervals during which the four classes of *actin(RNAi) *phenotypes were observed in the early embryo for this batch of dsRNA. Average maximum intensities and standard deviations are based on cumulative data from 3–5 gonads and three sampled areas per gonad (i.e. 9–15 data points) for each timepoint. Numbers on the vertical axis represent units of intensity as measured by Openlab 3.1.7 software. (C) Time-course depletion of actin and requirements for early embryogenesis. Each row, labeled by class, shows DIC images from a time series analysis spanning from just after the completion of meiosis to the second mitotic division. For each timepoint, hallmark events in wild-type (top row) are listed above each column. *actin(RNAi) *Class I phenotypes (n = 11 embryos): Loss of cortical contractions, no pseudocleavage, and synchronous second cell division. *actin(RNAi) *Class II phenotypes (n = 12): Pronuclei meet more centrally, loss of spindle displacement, and parallel and synchronous second cell division. Note the increase in distance between the paternal nucleus and the posterior cortex. *actin(RNAi) *Class III phenotypes (n = 12): First cell division is incomplete with nuclear reformation close to contractile ring, and multiple nuclei in subsequent divisions. *actin(RNAi) *Class IV phenotypes (n = 14): Failure to extrude polar bodies, loss of cytokinesis and multiple nuclei. Arrows point to polar bodies (where observed). Scale bar is 10 μm.

To ask if different processes in the early embryo that depend on an intact actin cytoskeleton are differentially sensitive to the amount of available actin, we performed a time-course analysis of early embryogenesis after injection of dsRNA targeting all actin genes using *act-1(RNAi) *(see Materials and Methods, Figure 1, and Additional File 1: Supplementary Figure 1). To verify and quantify the depletion of actin, we used an anti-actin antibody to detect the amount of protein in the gonad over time. We observed that protein levels were reduced by about half at approximately 6 hours post-injection, declined sharply between 7–8 h, and finally tapered off (Figure 1A and 1B). We will refer to the embryos produced by treated animals as *actin*(*RNAi*) embryos. Analysis of *actin*(*RNAi*) embryos at distinct time points revealed a phenotypic series analogous to what might be seen if we had an allelic series for a family of redundantly functioning genes (Figure 1B and 1C).

**Figure 2:**
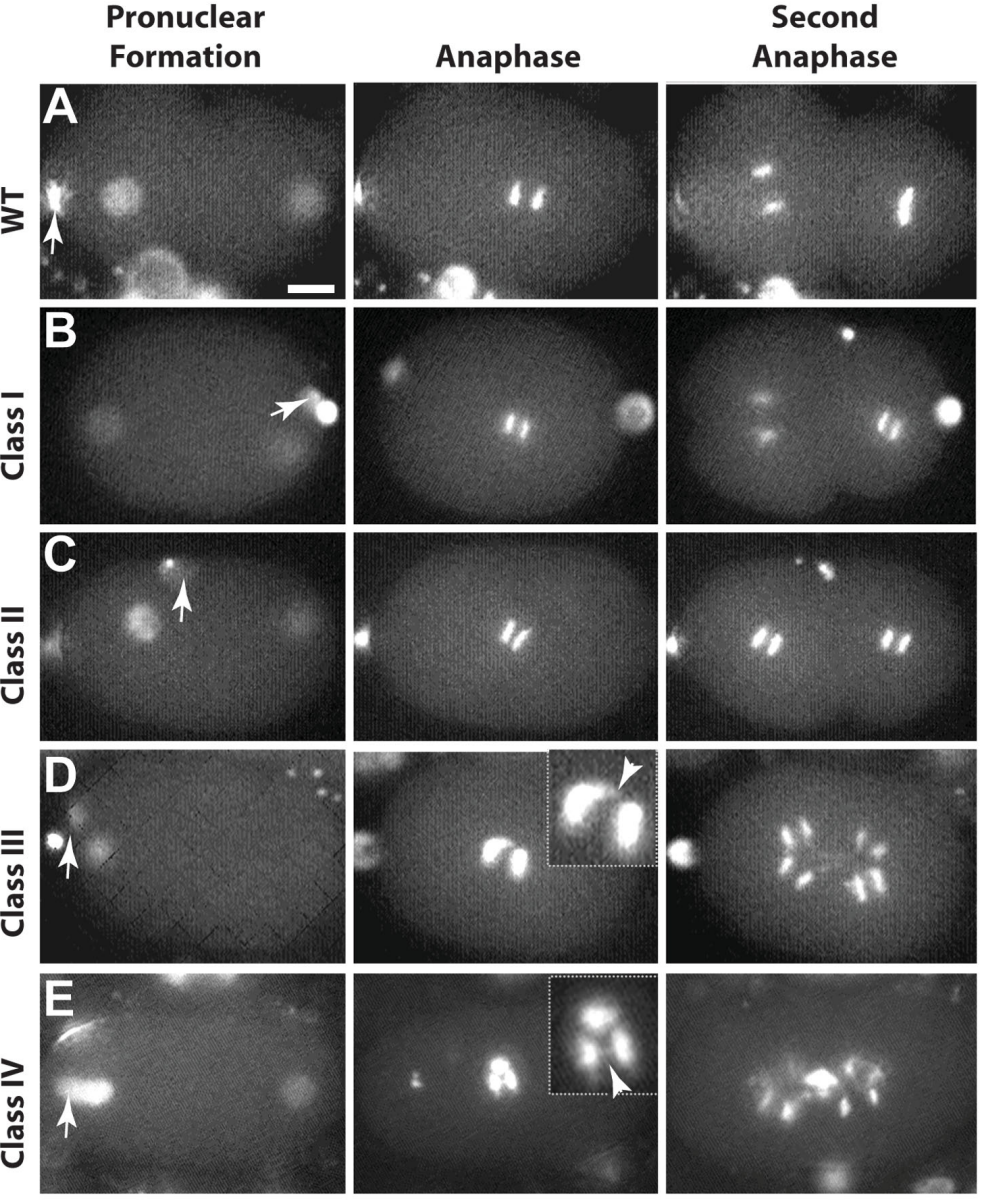
***actin(RNAi) *affects extrusion of polar bodies, chromosome segregation, and relative timing and rotation of mitotic spindles**. (A) In wild type, both polar bodies are extruded at the anterior end of the embryo (arrow indicates second polar body). Anaphase during the second round of mitosis occurs asynchronously, with the larger AB cell dividing first. The spindle in the P1 cell rotates and divides later and perpendicularly to the spindle in AB. (B) Class I (n = 5) embryos depleted of actin show synchronous second anaphase spindles. This is a unique case in which the polar bodies appear at the posterior pole of the embryo, indicating a reversal of polarity or that they have migrated with the secretion of the eggshell. (C) Class II (n = 5) embryos show anaphase spindles in the second division that are both synchronous and oriented parallel to each other. (D) Class III (n = 4) embryos show chromosome segregation and spindle elongation defects at anaphase in the first and second divisions. (E) Class IV (n = 4) embryos fail to extrude both polar bodies, which remain attached to the maternal pronucleus. Chromosome segregation and spindle elongation defects can also be seen (insets in D and E). Arrows in A-E point to polar bodies. Arrowheads in insets (D and E) point to chromatin bridges. Scale bar is 10 μm.

The wild-type (WT) embryo undergoes a series of canonical events that are easily observable using time-lapse DIC microscopy (Figure 1C, Additional File 4: Movie 1). We review them here and compare them with events that occur in *actin*(*RNAi*) embryos (Figure 1C, Additional Files 5, 6, 7, 8 Movies 2–5). After the completion of meiosis and the extrusion of two polar bodies, approximately 25 minutes after fertilization, the maternal and paternal pronuclei form at anterior and posterior ends, respectively. Concurrently, the cortex of the embryo undergoes intense contractions that cease at the posterior end of the embryo when the paternal pronucleus closely associates with it. These contractions culminate in a deep invagination near the middle of the embryo referred to as the pseudocleavage furrow. At this time, the maternal pronucleus migrates toward the paternal pronucleus. The pseudocleavage furrow relaxes as the pronuclei meet in the posterior half of the embryo. The pronuclei do not immediately fuse upon meeting, but move together toward the middle and rotate, such that the division axis is established perpendicular to the long axis of the embryo before nuclear envelope breakdown. The spindle is displaced at anaphase to the posterior of the embryo, giving rise to two daughter cells of different size. These cells have distinct fates giving rise to different parts of the adult worm. The distinct identities of these cells are characterized by the way they divide in the second round of mitosis: they undergo mitosis at different times, with the larger anterior AB cell dividing before the smaller posterior P1 cell, and their division axes are perpendicular to each other (Figure 1C, Figure 2A, Additional File 4: Movie 1).

The effect of *actin(RNAi) *on these canonical early embryonic events depends on the time elapsed between injection of dsRNA and initiation of embryogenesis, due to a progressive depletion of actin over time in the treated animal (Figure 1). Eventually, *actin(RNAi) *leads to a drastic reduction of fertilized embryos, approximately 12–15 hours after injection; in contrast, control animals produce fertilized embryos for at least twice as long (see Additional File 2: Supplementary Figure 2; [18]). These results are consistent with a recent analysis showing that the lateral plasma membrane collapses in gonads cultured with high concentrations of actomyosin inhibitors [36], which could lead to disruption of oocyte integrity and fertilization. Thus, the existence of any embryos indicates that oocyte integrity is maintained by residual actin activity, and therefore represents a hypomorphic condition. Prior to this study, the only hypomorphic phenotypes reported in *actin(RNAi) *embryos, as part of a large scale study, were failure to extrude polar bodies, loss of spindle displacement, and loss of cytokinesis [18]. By selecting embryos for analysis from RNAi-treated animals in a carefully timed way, we were able to observe a reproducible series of defects that we could calibrate for each batch of dsRNA (see Materials and Methods). We grouped embryos showing similar ranges of progressively more severe defects into four phenotypic classes (Classes I-IV; Figures 1C and 2). Specific phenotypes appearing together within a class thus represent processes that require similar levels of actin in the cell. The range of phenotypes observed in the four phenotypic classes is summarized in Additional File 3: Supplementary Table 1.

Class I defects (Figures 1C (n = 11) and 2B (n = 5), Additional File 5: Movie 2) highlight the processes that are affected by the mildest reduction in available actin, and thus require the highest levels of actin. In the first cell cycle, cortical ruffling and pseudocleavage were absent. Spindle movements in P0 appeared normal and the first cell division was asymmetric, whereas the AB and P1 cells divided in the correct orientation but with abnormally synchronous timing. Thus, Class I defects appear to separate two processes, spindle orientation and timing of the second division, which are thought to be coupled and to depend on proper polarity establishment in the one-cell stage. In Class II embryos (Figures 1C (n = 12) and 2C (n = 5), Additional File 6: Movie 3), the paternal pronucleus failed to maintain its close association with the posterior cortex, causing the pronuclei to meet more centrally. After nuclear fusion, the spindle failed to move toward the posterior prior to cytokinesis, giving rise to more symmetric daughter cells than in WT. Finally, the second mitotic cell divisions were both synchronous and oriented parallel to each other, transverse to the AP axis (Figure 2C). Class III embryos (Figures 1C (n = 12) and 2D (n = 4), Additional File 7: Movie 4) began to show cytokinesis defects, as well as disorganized furrows during subsequent cellular divisions that gave rise to polynucleated cells. Nuclear reformation occurred close to the contractile ring, an indication of reduced elongation of the central spindle during anaphase B. Severely depleted Class IV embryos (Figures 1C (n = 14) and 2E (n = 4), Additional File 8: Movie 5) failed both to extrude both polar bodies and to execute cytokinesis, and contained small karyomeres. Interestingly, even among the most strongly affected embryos, the rotation of the spindle in P0 occurred normally.

The reformation of the nuclei near the cell division remnant and the presence of karyomeres in *actin*(*RNAi*) embryos suggested that they might also experience chromosome segregation defects. To further explore the effect of actin depletion on chromosome segregation, we analyzed the same time points in a line expressing a GFP-histone fusion (GFP::HIS) [37], which reveals chromosomal movements and dynamics. In addition to confirming the orientation of mitotic chromosomes and cell cycle timings, we also observed that anaphase figures showed partially decondensed chromosomes, lagging chromosomes, and chromosome bridges in Class III and IV embryos (Figure 2D and 2E). These data indicate that severe actin depletion interferes with the proper resolution of chromosomal pairing and/or movement of chromosomes to the spindle poles.

### Cortical F-actin undergoes three phases of morphological transitions in the one-cell embryo

**Figure 3:**
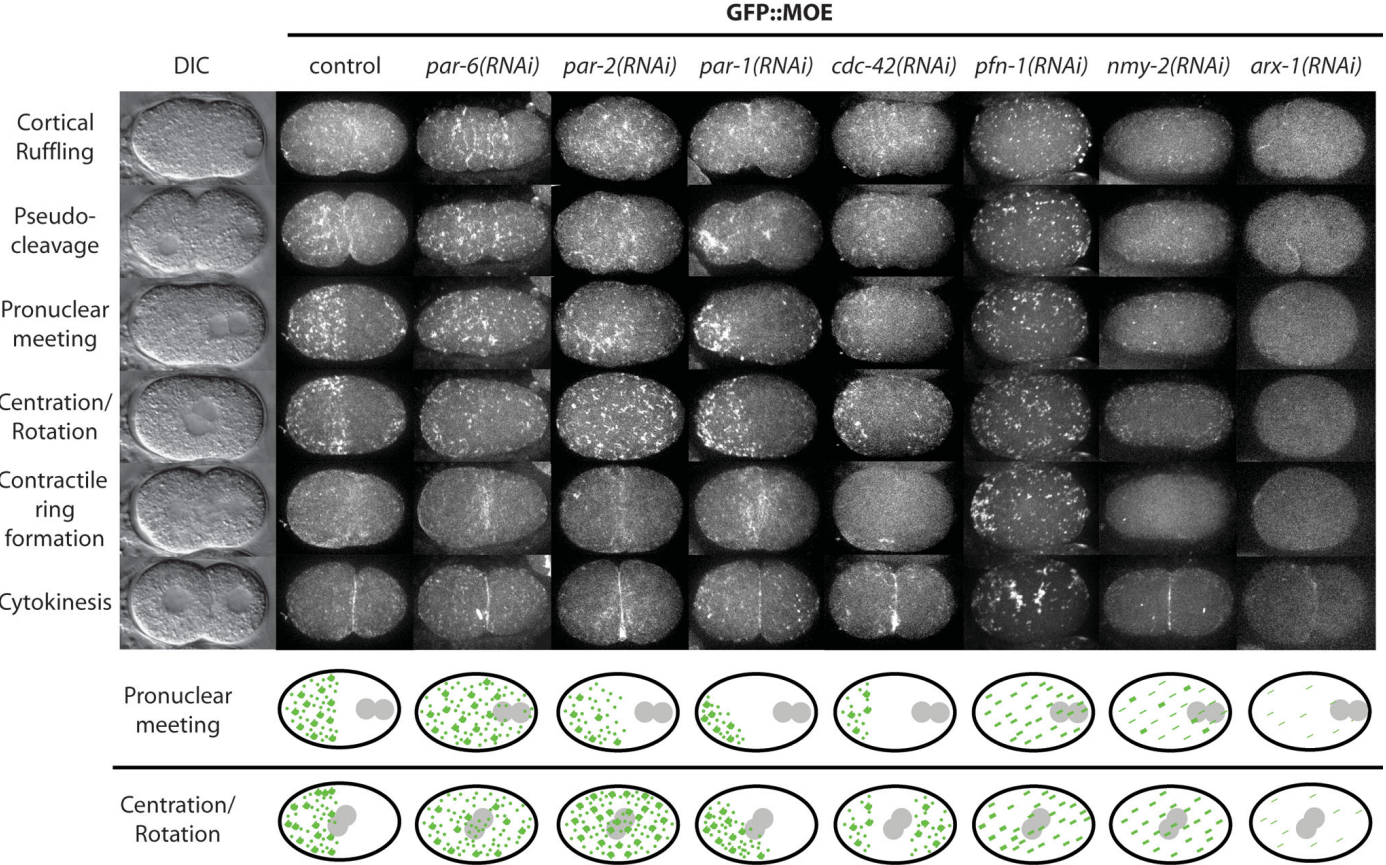
**Polarity determinants and F-actin regulators affect F-actin dynamics during early embryogenesis**. Columns show successive images from time-series analyses, from pronuclear formation to first mitotic division. Labels for each row of micrographs indicate hallmark events in WT at corresponding timepoints. Wild-type: F-actin becomes enriched in the anterior half of the embryo during the first cell division; maximum enrichment occurs after pronuclear meeting. Note the change from F-actin meshwork to discrete foci. *par-6(RNAi) *(n = 8): Initial clearing of the posterior end is lost by the time of pronuclear centration and rotation. The cell undergoes an equal cell division. *par-2(RNAi) *(n = 5): An initial displacement of F-actin occurs as in WT, but after pronuclear meeting F-actin begins to accumulate at the posterior. *par-1(RNAi) *(n = 11): F-actin becomes hyper-asymmetrically enriched, occupying approximately one-quarter the length of the embryo. No F-actin accumulates at the posterior. *cdc-42(RNAi) *(n = 6): F-actin progressively becomes asymmetrically distributed until pronuclear meeting, at which time F-actin begins to accumulate at the posterior. F-actin forms a weak filamentous network at the cortex and few foci are observed. *pfn-1(RNAi) *(n = 6): Only discrete foci are observed at the cortical surface of the embryo. These foci appear to move passively toward the anterior until pronuclear meeting, at which point foci begin to agglomerate into larger foci that disperse throughout the entire embryo cortex. Cytokinesis does not occur. *nmy-2(RNAi) *(n = 4): Weak depletion of NMY-2 disrupts the actomyosin network at the cortex. Very weak filamentous structures are observed with few thick foci forming at pronuclear meeting. The contractile ring is slow to form and eventually disintegrates (data not shown). *arx-1(RNAi) *(n = 4): Almost no detectable F-actin structures are observed at the cortical surface. F-actin was recruited to the contractile ring and cytokinesis occurred as in WT. The last two rows depict actin morphology at the cortex (in green) during pronuclear meeting and pronuclear centration and rotation (pronuclei in gray). Scale bar is 10 μm.

To visualize actin dynamics *in vivo *in the early embryo we fused GFP with the *D. melanogaster *Moesin F-actin binding domain [38]. This GFP::MOE marker has been shown to faithfully report the distribution of microfilaments in both *D. melanogaster *[39] and *C. elegans *[38]. All the structures that we observe with our GFP::MOE line are therefore F-actin-rich structures decorated with GFP molecules. Previous analyses using phalloidin-stained fixed specimens [19,20] or *in vivo *visualization of NMY-2::GFP dynamics [23] during the first cell cycle of embryogenesis have shown that actomyosin forms a cortical lattice that appears to contract asymmetrically to the anterior half of the embryo [23]. GFP::MOE embryos similarly show a dynamic meshwork of cortical actin fibers and dense focal accumulations, as well as numerous small punctate structures. These structures all become enriched to the anterior with similar kinetics as NMY-2::GFP. However a persistent collection of small actin-rich puncta remains at the extreme posterior throughout the first cell cycle. The asymmetric redistribution of microfilaments does not appear to result from collapse of the meshwork under tension; rather, the dynamic formation and dissipation of F-actin structures that we observe in the anterior indicates that microfilaments are in rapid flux. This suggests that the capacity for filament assembly becomes segregated to the anterior domain while assembly is reduced in the posterior. At pronuclear meeting, the fibrous actin meshwork revealed by GFP::MOE transforms into a collection of discrete puncta in the anterior half of the embryo that persists until just prior to contractile ring formation, at which point cortical F-actin rapidly dissipates (Figure 3, Additional File 9: Movie 6).

Thus, there appear to be three phases involving a major reorganization of the cortical actin cytoskeleton that are coordinated with the cell cycle in the one-cell embryo: first, the asymmetric redistribution of F-actin structures to the anterior (we refer to this as "Phase I"); second, concomitant with pronuclear meeting and centration, a shift in the overall morphology of predominant cortical F-actin structures from a dynamic meshwork to discrete puncta ("Phase II"); and finally, a rapid clearance of cortical F-actin in anticipation of cytokinesis ("Phase III").

### The proper anterior redistribution of microfilaments depends on PAR proteins

Previous work has shown that the distribution of microfilaments to the anterior half of the embryo, as visualized in fixed preparations, is affected by mutations in *par *genes [20]. To investigate this relationship *in vivo*, we performed time-lapse microscopy of GFP::MOE embryos depleted of different PAR proteins using RNAi. For all *par *genes we analyzed (*par-6*, *par-2*, and *par-1)*, we compared RNAi phenotypes with published phenotypes for the strongest reported genetic alleles to confirm that the phenotypes we obtained are as strong as the genetic alleles. Upon RNAi of PAR-6, a member of the anterior PAR complex, F-actin initially assembles on the cortex as a filamentous meshwork, similar to WT (n = 8; Figure 3, Additional File 10: Movie 7). *par-6(RNAi) *embryos show a weak clearing of the posterior domain prior to pronuclear meeting. The actin meshwork in *par-6(RNAi) *embryos resolves into discrete puncta during Phase II at a similar time as in WT; however, these puncta do not concentrate in the anterior as in WT, but are distributed throughout the cortex (n = 8; Figure 3). Thus any weak early asymmetries are lost by pronuclear centration and rotation. Similarly, *par-2*(*RNAi*) embryos show an initial weak depletion of cortical F-actin from the posterior (Phase I), but F-actin becomes uniformly distributed throughout the cortex and resolves into discrete punctate structures by the time pronuclei rotate and centrate (n = 5; Figure 3, Additional File 11: Movie 8). In comparison with *par-2(RNAi)*, actin structures appear less prominent at the cortex in *par-6(RNAi) *embryos. This is most notable during Phase II in *par-6(RNAi) *embryos, when PAR-2 is uniformly distributed throughout the cortex) [10]. Cortical NMY-2::GFP is similarly reduced in *par-3(RNAi) *but not *par-3; par-2(RNAi) *embryos [23]. In *par-1*(*RNAi*) embryos, abnormal accumulations of actin appear in the meshwork during Phase I, and by pronuclear meeting F-actin becomes hyper-asymmetrically enriched in the anterior (n = 11; Figure 3, Additional File 12: Movie 9). As pronuclei centrate and rotate, a morphological transition to discrete puncta is clearly discernable (Phase II) and the posterior F-actin boundary expands slightly toward the midline of the A-P axis (Figure 3). PAR-1 might thus be required to set up the border of the anterior F-actin domain, similar to its role in establishing the PAR boundary [6,10,20]. In all three RNAi treatments, actin-rich puncta are evident at the extreme posterior of the embryo during Phase I, similar to WT. We conclude from these results that PAR-2 and PAR-6 proteins are primarily required to constrain actin-rich structures to the anterior half of the embryo, whereas PAR-1 contributes to the proper coordination of actin dynamics and establishment of the correct boundary of the anterior F-actin domain.

The diversity of actin-rich cortical structures and regulated transitions in their morphology led us to investigate the role of F-actin regulators on their dynamics. Regulators of the actin cytoskeleton have been shown to influence the localization of PAR proteins in *C. elegans *and other organisms [6,24]. Severe depletion of CDC-42, which binds PAR-6 [40], leads to sterility and osmotic sensitivity, and the actin cytoskeleton fails to assemble on the cortical surface (data not shown) [24,41,42]. Less severe depletion of CDC-42 does not affect the initial polarization of F-actin to the anterior during Phase I, but by pronuclear meeting actin becomes hyper-asymmetrically concentrated in the anterior (n = 6; Figure 3, Additional File 13: Movie 10), similar to *par-1*(*RNAi*) embryos. Unlike *par-1*(*RNAi*), however, this hyper-asymmetry persists throughout Phase II, and the domain of F-actin-rich puncta at the extreme posterior appears to be expanded relative to WT during Phase II. Overall, the association of F-actin structures with the cortex appears weaker than in WT, and the contractile ring is very faint. Therefore, CDC-42 is needed to maintain the initial asymmetric distribution of F-actin and for a strong association of F-actin with the cortical surface.

### Actin-binding proteins and nucleators of actin affect the morphology of cortical microfilaments

Profilins are actin-binding proteins that also interact with other regulators of the actin cytoskeleton and can affect actin dynamics in a variety of ways depending on the molecular context (reviewed in [43]). *C. elegans *contains three profilins with similar biochemical properties that all behave as classical nonvertebrate profilins [44]. Only one of these, PFN-1, is essential for embryogenesis, where it is required for cytokinesis and polarity in the one-cell embryo and is also required for the proper localization of F-actin and NMY-2 to the cortex [16]. To investigate profilin's role in polarity and its effect on cortical microfilaments in more detail, we analyzed embryos depleted of PFN-1 by time-lapse microscopy. We found that *pfn-1(RNAi) *prevented the appearance of an actin-rich meshwork at the cortex, while actin-rich puncta appeared much earlier and moved more freely around the cortex than in WT (n = 6; Figure 3, Additional File 14: Movie 11). These puncta began to coalesce into large actin-rich clumps between pronuclear meeting and centration (when the Phase II transition normally occurs in WT). Cytokinesis never occurred, although some residual contractile activity could be observed on the cortex during mitosis (Additional File 14: Movie 11 shows a striking ring-like formation of cortical actin-rich structures that appears to contract into a single aggregate). These observations indicate that the early embryo contains two genetically separable populations of cortical actin-based structures, a fibrous meshwork and distinct punctate accumulations, and that profilin appears to be required exclusively for the assembly of filaments that form the meshwork. Profilin is also required to correctly position and coordinate contractile activity required for cytokinesis with the mitotic spindle, as has been observed previously in *C. elegans *and other systems [45].

NMY-2 encodes the heavy chain of a non-muscle myosin motor protein that is required for cytokinesis and has been shown to affect polarity in the *C. elegans *one-cell embryo [13]. *nmy-2(RNAi) *embryos can be classified by the severity of phenotypes resulting from variable depletion of NMY-2 activity, from weak to strong) [10,13]. Weak "Class II" NMY-2 depletion gives a PAR-like phenotype, and can thus be used to study the connection between polarity and actin. GFP::MOE dynamics revealed that the overall organization of cortical F-actin structures was disturbed in *nmy-2(RNAi) *Class II embryos and that association of microfilaments with the cortex appeared to be reduced. The contractile meshwork was absent and no asymmetric contraction occurred (Phase I); punctate accumulations were evenly distributed throughout the cortex at pronuclear meeting, and the morphological transition to Phase II did not occur (n = 4; Figure 3, Additional File 15: Movie 12). The cortex cleared prior to contractile ring formation as in WT, but the first division was symmetric and the spindle failed to rotate in P1 (a clear *par*-like phenotype). Thus, like PAR proteins, the asymmetric localization of actin during the first cell cycle depends on NMY-2 function. These data demonstrate that the proper assembly and organization of a variety of actin-based cortical cytoskeletal structures depends on myosin function.

The Arp (actin-related protein) 2/3 complex promotes rapid actin polymerization in response to various signals and results in the formation of branched filaments, which allow for cytoskeletal reorganization in the cell [5,46-49]. In *C. elegans*, depletion of either ARX-1 (Arp2) or ARX-2 (Arp3) leads to embryonic lethality; while no early embryonic phenotypes have been reported, ventral closure and postembryonic defects have been observed [50,51]. Disruption of the Arp2/3 complex by depletion of ARX-1 prevented the formation of an F-actin meshwork at the cortex; a few transient F-actin structures were observed, but no foci formed upon pronuclear meeting (n = 4; Figure 3, Additional File 16: Movie 13). Despite the lack of F-actin enrichment in the anterior, the cell was able to undergo an asymmetric cell division, indicating that other factors required for the establishment of polarity and the boundary between anterior and posterior cortical domains were unaffected by depletion of ARX-1. F-actin was properly recruited to the contractile ring and cytokinesis occurred as in wild type.

### The *C. elegans *embryo contains highly dynamic actin-rich "comets"

**Figure 4:**
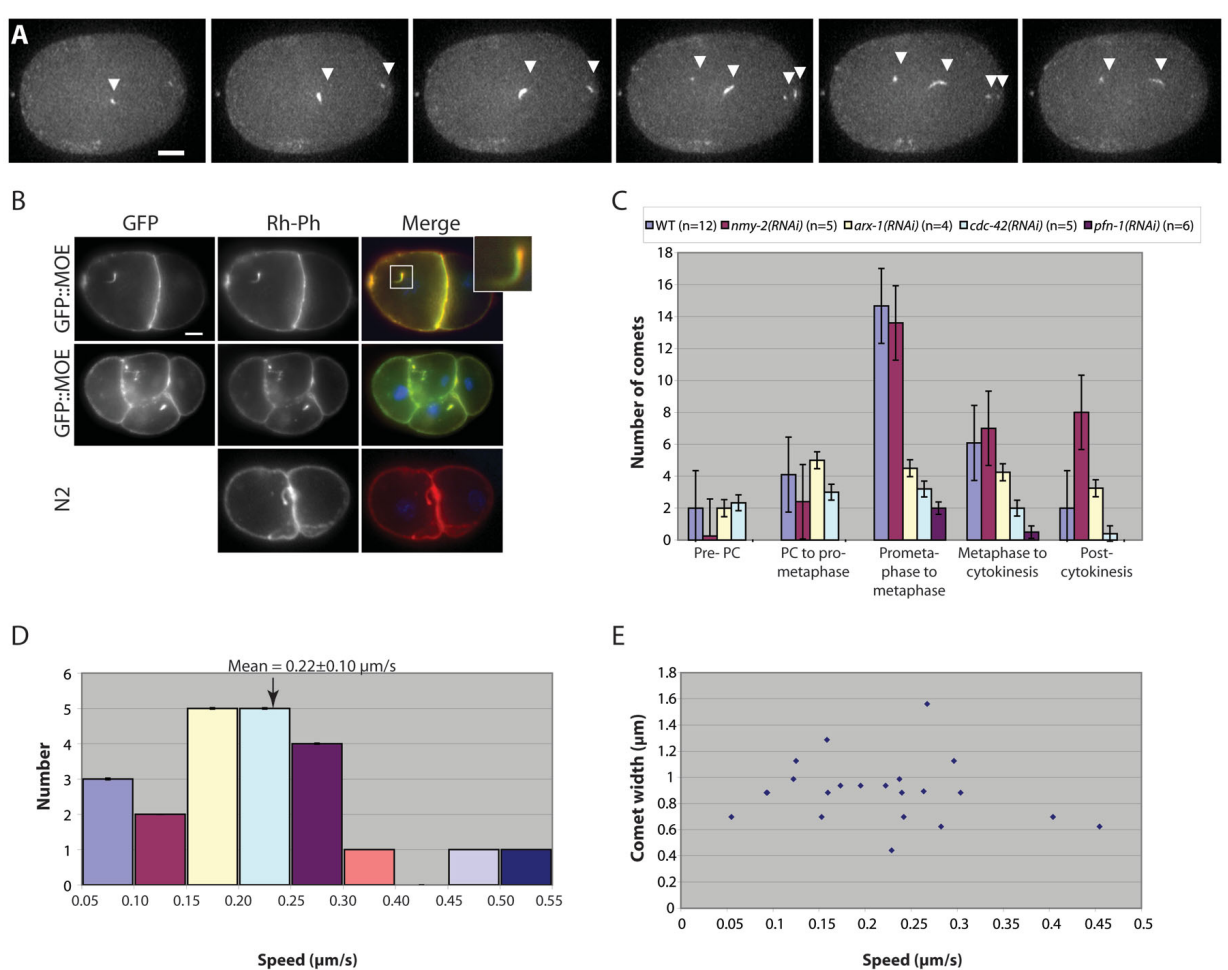
**Actin comets are dynamic and cell cycle-dependent**. (A) Comets vary in speed, length, width, and direction of movement. Six consecutive frames show the 3D-Max Projection of 8 sections (1 μm Z-step at 6 s intervals) through the top half of an embryo at pronuclear meeting. (B) Rh-phalloidin labels actin comets and colocalizes with GFP::MOE. Fixed 2- and 4-cell embryos from wild-type N2 and GFP::MOE transgenic animals (inset, magnified image of one comet tail). No DAPI staining is observed at the tips of comet tails. (C) The number of actin comets peaks just prior to prometaphase of the first cell cycle in WT and is influenced by RNAi of several actin-binding proteins. Actin comets were counted in the top half of the embryo during five consecutive time intervals of 3–4 minutes each, bounded by the following landmarks: 3 minutes before pseudocleavage (PC), pseudocleavage, prometaphase, metaphase, completion of cytokinesis, cytokinesis + 3 min. RNAi of Arp2/3 (ARX-1), CDC-42, and profilin (PFN-1), but not non-muscle myosin (NMY-2), significantly reduces the maximum number of comets. (D) Distribution and mean speed (0.22 ± 0.10 μm/s) of comet tails in the one-cell embryo. Measured speeds of individual comets (n = 21) were binned into 0.05 μm intervals. (E) A scatterplot of tail width and speed shows that tail width and speed are not correlated. Scale bars in A and B are 10 μm.

In the course of characterizing actin dynamics in the GFP::MOE strain, we were struck by the presence of "comet tails" that moved rapidly through the cytoplasm and near the cortex in the one-cell embryo. Actin-rich comets have been described in association with both endocytic vesicles [5,26-28,30] and intracellular pathogens such as *Listeria, Shigella*, and *Rickettsia *(reviewed in [5,31-33]). In *C. elegans*, we noticed that the comets have no consistent direction – often appearing as curvy or curly structures – and that they vary in length, speed and number throughout the first cell cycle (Figure 4, Additional File 17: Movie 14). We also observed comets in developing and maturing oocytes and in later stages of embryogenesis (data not shown). The observation of similar unknown structures has been reported in fixed samples [19], but their dynamic nature has not previously been described. To confirm that actin comet tails were not artifacts of the GFP::MOE transgenic line, we visualized F-actin in fixed WT and GFP::MOE embryos with rhodamine-conjugated phalloidin (Rh-phalloidin). Rh-phalloidin clearly labeled comet tails in both lines, and co-localized with GFP antibodies in the GFP::MOE line (Figure 4B). DAPI did not stain the heads of the comet tails, dispelling the notion that they are associated with an intracellular bacterial pathogen (Figure 4B).

To begin to elucidate the potential role of actin comets, we characterized their behavior. Since these comets are more numerous during the first cell cycle, we quantified the number of comets present during this time and observed that the number of comets peaks around prometaphase (Figure 4C). Additionally, during the period from pronuclear meeting to prometaphase, the comets appear to coalesce around the pronuclei and sometimes to associate with the centrosomes (Additional File 11: Movie 8). This suggests that an accumulation of any cargo propelled by the comets may ultimately be required at some location near or defined by the nuclei and/or mitotic spindle. We measured speeds of actin comets in *C. elegans *and found that they travel at an average of 0.22 ± 0.10 μm/s (Figure 4D), similar to the mean speed and range of endocytic vesicles in transfected rat basophilic leukemia (RBL) cells (0.24 ± 0.10 μm/s) [27]. Although we observed that some comets were thicker than others, we found no correlation between their widths and speed (Figure 4E). We note that the trajectories and speeds of actin comets are quite distinct from those observed for an early endosome marker in the early embryo [34]; thus we believe they represent a different population of cellular structures.

We next asked if the actin comets were dependent on known regulators of F-actin assembly such as CDC-42, ARX-1 (Arp2), and PFN-1, which are required for the proper formation of actin comets in other systems. The Arp2/3 complex nucleates microfilaments, and CDC-42 promotes Arp2/3 activity by stimulating nucleation promoting factors (NPFs) that interact with Arp2/3 (reviewed in [5,31-33]). Depletion by RNAi showed that all three actin regulators are required to achieve the maximal number of comets observed at prometaphase in WT (Figure 4C). Depletion of PFN-1, which promotes polarized filament growth and increases the rate of movement of comets in other systems [31,32], caused the most dramatic reduction in the basal number of comets, which rose only slightly at prometaphase (Figure 4C). We cannot distinguish between the possibilities that further reduction of these proteins would lead to complete loss of comets, or that other cellular factors may be able to promote a basal level of filament nucleation in these comets. In contrast, depletion of PAR-6 (data not shown) or NMY-2 (Figure 4C) had no significant effect on either basal or maximal comet numbers, although in *nmy-2(RNAi) *embryos comets persisted well beyond telophase, particularly when the contractile ring was weak or failed to form (Figure 4C and data not shown).

We noticed no qualitative change in the speed or length of the comet tails in *arx-1(RNAi)*, *cdc-42(RNAi), pfn-1(RNAi)*, or *nmy-2(RNAi) *embryos (data not shown). We conclude that actin-rich comets in the *C. elegans *one-cell embryo are influenced by a cell cycle-dependent process that is coordinated with the activity of actin cytoskeletal regulators and that their speed is consistent with a potential role in endocytosis, but further characterization is needed to determine their precise role during development.

## Discussion

### Actin-dependent processes in the early embryo are differentially affected by depletion of actins by RNAi

Since actin is required for many diverse processes in the cell, deciphering its specific contribution to different cellular processes during development is challenging. By performing a time-course depletion of multiple actin isoforms simultaneously, we have described four classes of increasingly severe phenotypic responses in the early embryo. We have shown that among the processes most sensitive to actin depletion are cortical ruffling, pseudocleavage, and establishment of polarity. Progressively more severe depletion of actins led to reduced elongation of the central spindle, failure to displace the spindle toward the posterior, aberrant coalescence of telophase chromosomes, failure to extrude polar bodies, cytokinesis failure, and the appearance of chromatin bridges during mitosis. Eventually, sterility ensues, indicating a requirement for actin in oogenesis [36] and potentially in fertilization. We never observed defects in centrosome duplication or separation, or in rotation of the pronuclei in the one-cell embryo, indicating that microtubule-dependent processes are largely unperturbed by defects in the actin cytoskeleton. However, defects in spindle positioning and elongation during anaphase suggest that microtubule interactions with the cortex are perturbed by disruption of the actin cytoskeleton. These analyses allow us to differentiate processes that have a strict requirement for actin (cortical contractility, pseudocleavage, and polarity) from those that can take place in the presence of a lower level of actin (spindle displacement and elongation, polar body extrusion, cytokinesis, and fertilization) or those that appear to be completely independent of actin at these stages (nuclear migration, spindle rotation, and centrosomal duplication and separation).

### Microfilament dynamics during early embryogenesis

We have constructed a GFP::MOE line that enables observation of the dynamic nature of the actin cytoskeleton during early development in *C. elegans*. Using this GFP::MOE line, we have examined the dynamics of actin during the first cell division at high resolution and describe in detail the dynamic process in which F-actin becomes asymmetrically distributed to the anterior half of the embryo during the first mitotic cell division. Our observations clearly reveal three distinct phases of cortical F-actin dynamics during the first cell cycle: I) an anterior redistribution of a dynamic meshwork of fibers and foci corresponding to the period of cortical flow, II) a subsequent morphological transition to punctate structures in the anterior, and III) a global clearing from the cortex prior to assembly of the cytokinetic furrow. This final clearing of cortical F-actin in anticipation of cytokinesis (Phase III) has not been previously described.

Studies using either fixed specimens stained with phalloidin derivatives [19,20] or *in vivo *imaging with NMY-2::GFP [23] or GFP::MOE [24] to visualize the actomyosin network have described an asymmetric distribution of both F-actin and myosin to the anterior during the first cell cycle. We also observe that F-actin becomes asymmetrically enriched in the anterior half of the embryo (Phase I), concomitant with the anterior PAR proteins and NMY-2) [10,23,24]. However, in contrast to the NMY-2 lattice at the cortex, which visibly contracts toward the anterior, the cortical F-actin meshwork appears to contain dynamic fibers that continuously form and disappear, which become progressively restricted to the anterior half of the embryo (Figure 3, Additional File 9: Movie 6). As discussed below, this anterior redistribution depends on actin regulators and PAR proteins.

At pronuclear meeting, the morphology of actin structures transforms from a fibrous meshwork into a collection of distinct punctate structures (Phase II). This transition coincides with the change of morphology of cortical NMY-2::GFP structures from large dynamic aggregates to a field of small puncta [23], and a transition in the appearance of anterior PAR-6 from a punctate to smooth morphology [24]. This morphological transition represents a second phase of actin reorganization.

### Establishment of polarity

During the establishment of cortical polarity, the observation that filaments in the anterior domain apparently undergo rapid turnover suggests that some factor(s) required for filament nucleation and/or elongation becomes restricted to the anterior, such that filaments are rapidly lost in the posterior during the period of cortical flow. This observation is consistent with the finding that at the onset of polarity ECT-2, a putative guanine exchange factor and activator of RHO-1, becomes excluded from the posterior cortex in the vicinity of the sperm pronucleus, and this is dependent on an intact centrosome [24]. Both ECT-2 and RHO-1 are required for the localization of actin to the cortex and for polarization of the embryo [24]. Interestingly, the introduction of sperm-associated RhoGAP CYK-4 (which inactivates RHO-1), is also required for polarity, but *cyk-4(RNAi) *embryos retain cortical contractility [25]. Thus while contractile myosin activity is important for establishing polarity, other factors regulated by Rho signaling must also play a central role in this process. Taken together, these results suggest that microfilaments in the cortical meshwork are normally in flux, that these filaments rapidly disassemble in the posterior after onset of polarity due to the depletion or inactivation of factors capable of sustaining filament nucleation and dynamics, and that these factors are regulated downstream of RHO-1.

In other systems, Rho family proteins regulate both of the primary actin nucleation mechanisms in the cell, the Arp2/3 complex and Formin Homology (FH) proteins [2]. Arp2/3 appears to be dispensable for polarity in the *C. elegans *embryo [16] but is necessary for the formation of the actomyosin meshwork at the cortex (Figure 3). Formins recruit profilin to rapidly extend the free ends of uncapped actin filaments (reviewed in [52,53]). Our observations show that depletion of *C. elegans *PFN-1 leads to a marked reduction in the number of actin comets in the cytoplasm (Figure 4C), prevents the formation of the fibrous meshwork observed in WT, and ultimately leads to loss of polarity and cytokinesis failure. *pfn-1(RNAi) *embryos initially display small punctate structures at the cortex that increase in number and begin to aggregate as the cell cycle progresses. The reduction of the actin meshwork but the retention of prominent actin-rich puncta upon depletion of PFN-1 suggests that the populations of microfilaments in these two types of structures are distinct, and that PFN-1 contributes primarily to the elongation or turnover of filaments in the meshwork. The existence of different subpopulations of cortical microfilaments would be consistent with work in other systems showing that different types of actin structures are nucleated and organized by different combinations of actin-binding proteins [40,54-56].

Our observations of highly dynamic actin fibers in WT and their loss in *pfn-1(RNAi) *embryos, combined with data demonstrating that polarization depends on RHO-1 activity, suggest a mechanism for breaking the actomyosin symmetry in response to the polarization cue. Like PFN-1, the FH protein CYK-1 is required for the maintenance of microfilaments on the cortex, and PFN-1 binds to CYK-1 [16]. Profilin retains its cortical localization even when microfilaments are eliminated in the embryo by treatment with Latrunculin A [16]. Together these data suggest the possibility that filaments comprising the actin meshwork in the *C. elegans *early embryo are nucleated and rapidly extended primarily by CYK-1 working together with PFN-1, and that this activity is regulated by RHO-1 and its effectors. Down-regulation of RHO-1 activity in the posterior would lead to a localized loss of this nucleation/elongation activity and consequent rapid disassembly of actin filaments. This localized absence of filament integrity in the posterior would in turn lead to cortical flow, mediated by the migration of cortical contractile activity toward the anterior as the uniform meshwork maintaining balance in contractile forces is lost. Additional factors could also contribute to this mechanism, as Rho family proteins in other systems are known to also regulate contractility and actin dynamics through myosin light chain kinase and cofilin [2]. Further investigation will be required to precisely define the mechanisms through which microfilament dynamics are regulated in the early embryo.

### PAR proteins and the cortical actin cytoskeleton

As previously described, the anterior PAR proteins are required for the proper localization and maintenance of F-actin in the anterior [20]. We also found that PAR-6 is needed for the proper segregation of F-actin to the anterior domain. We observed an initial weak clearing of actin in the posterior cortex that was lost by the time of pronuclear centration and rotation, at which time actin was distributed uniformly throughout the cortex. *par-2(RNAi) *resulted in a similar effect on the cortical actin cytoskeleton, including an initial asymmetry that was then lost. These results suggest that the PAR system is not required for breaking actin symmetry, but is required for reinforcement and maintenance of actin asymmetry, and that the anterior PAR complex and actin cytoskeleton are mutually dependent for the maintenance of polarity in the one-cell embryo. The PARs and the actin cytoskeleton may thus respond to the same initial cues that eventually lead to the establishment and maintenance of distinct anterior and posterior domains. This is consistent with previous reports providing evidence for positive feedback between the PAR system and the actomyosin cytoskeleton during the establishment and maintenance phases [23,24].

Our results suggest that PAR-1 and CDC-42 are needed to establish the proper boundary of F-actin distribution at pronuclear meeting, as depletion of these proteins causes a hyper-asymmetric enrichment of F-actin to the anterior. Loss of CDC-42 also allows some F-actin to begin to accumulate at the posterior pole by the time of pronuclear centration and rotation. Overall we observed fewer foci, and cortical fibers appeared fainter, in embryos depleted of CDC-42 as compared with WT, indicating that regulation of filament dynamics by CDC-42 contributes to general actin cytoskeletal organization in the early embryo. CDC-42 is required for the maximal cortical accumulation and maintenance of the PAR-6 domain [57]; thus a reduced level of cortical PAR-6 in *cdc-42(RNAi) *embryos could contribute to the observed reduction in the extent of the anterior F-actin domain. Severe depletions of CDC-42 caused embryos to become osmotically sensitive ~36 hours after injection and worms eventually became sterile, potentially revealing cross-talk between signaling pathways controlling the actin cytoskeleton and other cortical activities such as egg shell formation.

### Actin comets and their potential role in the cell

In this paper we describe the presence and dynamics of highly motile actin comets that have not been previously recognized in *C. elegans*. Similar comet tails have been observed in association with the movement of different types of endocytic vesicles and intracellular pathogens, which exploit the cell's cytoskeletal machinery to propel them through the cytoplasm and into neighboring cells (reviewed in [5,31-33]). The Arp2/3 complex has been implicated in the internalization of membranous organelles in *Dictyostelium*, budding yeast, *Xenopus *extracts and eggs, and mammalian cells. *In vivo *studies and *in vitro *reconstitution experiments have also established that the Arp2/3 complex is one of the primary cellular factors driving the propulsion of actin-rich tails. The Arp2/3 complex nucleates new actin filaments and cross-links newly formed actin filaments in a branched pattern, and is regulated by Rho family proteins and a variety of nucleation promoting factors (NPFs) (reviewed in [53]). Organelles and bacterial pathogens initiate filament assembly by stimulating Arp2/3 activity through a combination of such factors, and use polymerization at the rear surface to generate the propulsive force for intracellular movement.

To begin to address what the role of such comets could be in *C. elegans*, we characterized the dynamics and behavior of the actin comets in the first embryonic cell cycle, where they are most easily observed. We found that the speed at which actin comets move in the *C. elegans *early embryo (0.22 ± 0.10 μm/s) is very close to reported speeds for actin comet tails associated with endocytic vesicles in mammalian cells (0.24 ± 0.10 μm/s) [27]. We observed no correlation between the speed of the comets and their width (thickness), suggesting that the rate of movement is not limited by physical factors such as resistance to movement due to viscosity of the cytoplasm, but may be determined by molecular factors such as actin filament nucleation or polymerization rates. In support of this idea, the motility of *Listeria *occurs at the rate of actin polymerization and depends on the availability of renewable ATP and molecular tail components required for propulsion (such as actin and profilin) [58,59].

We found that the number of actin comets in the one-cell embryo is cell-cycle-dependent, peaking around prometaphase. Consistent with observations in reconstituted systems [31], NMY-2 appears to be dispensable for the formation and dynamics of comets in *C. elegans*. However partial depletion of ARX-1 (the Arp2 subunit of the Arp2/3 complex), CDC-42 (a GTPase that stimulates assembly of actin filaments nucleated by Arp2/3), or PFN-1 (which promotes polarized filament growth), led to a significant reduction in the peak number of actin comets. Similarly, in yeast, a temperature-sensitive mutation in the Arp2p subunit of the Arp2/3 complex – which is known to cause defects in endosome internalization but does not affect actin cables or trafficking of internalized endosomes [60] – led to a reduction in the number, but not the velocity, of retrograde actin patch movements away from the cell cortex [5]. Because we cannot completely deplete these proteins in the embryo by RNAi (more severe depletions lead to sterility and osmotic defects), it is possible that further depletion would lead to more severe effects. However, our results are consistent with the idea that Arp2/3 activity stimulates the nucleation of actin comets in *C. elegans *embryos. Studies performed *in vitro *have shown that profilins affect the rate of movement of actin tails but not their numbers [31,32]. In contrast, we noticed no significant change in the velocity of comets in PFN-1 depleted embryos, but the number of comets was drastically reduced at all stages of the cell cycle. Further work will thus be needed to understand the mechanisms by which actin filament dynamics are controlled in these structures and how this translates into propulsive force.

We do not yet understand the biological role fulfilled by actin comets in *C. elegans*. While the nature of their cargo is still unknown, they display similar morphological and dynamic characteristics with Arp2/3-dependent comet tails associated with endocytic vesicles in other metazoan cells. It thus seems likely that actin comets in the *C. elegans *embryo represent the movement of some type of endocytic vesicles. Based on their numbers and behavior, they appear to be distinct from early endosomes recently described in the *C. elegans *embryo, which are much more numerous on the cortex and move internally directly toward centrosomes at a rate consistent with dynein-mediated transport along microtubules [34]. The observations that few comets appear to be present at any one time, and that their numbers are coordinated with the cell cycle, suggests a highly specialized function. For example, actin comets might mediate the regulated internalization of specific cell-surface factors [61]. The precise roles that actin comets play in the one-cell embryo and whether they have additional roles in other cellular and developmental processes remain open questions.

## Conclusion

This study shows that progressive depletion of actins by RNAi can reveal processes during early embryogenesis with differing requirements for actin availability. Using a GFP marker to visualize F-actin dynamics *in vivo*, we reveal the dynamic behaviors that microfilaments exhibit during the first cell cycle of *C. elegans *embryonic development at a level of detail previously inaccessible due to the highly dynamic and transient nature of many of these events. We show how microfilaments are regulated during this time period using RNAi of PAR proteins and regulators of microfilament dynamics. Additionally, we describe for the first time the appearance and dynamics of actin comets during development.

## Methods

### *C. elegans *strains and GFP fusions

General methods for culturing and handling were carried out as previously described [62]. All experiments were carried out at 25°C unless otherwise indicated. The strains and genetic markers used were as follows: wild-type Bristol strain (N2) and *nnIs *[*unc-119(+) pie-1 promoter::gfp::Dm-moesin*^*437*–*578 *^(amino acids 437–578 of *D. melanogaster *Moesin)] (GFP::MOE) [38].

### RNAi

RNAi was performed by injection or soaking [63]. Clones were obtained from the genome-wide feeding library [50], verified by restriction digests, and PCR amplified with T7 primer. In some cases, gene-specific primers containing partial T7 polymerase promoter sequences were first used to amplify a portion of each gene from genomic DNA, followed by a second round of PCR with full T7 primer. PCR products were used as templates to make dsRNA using the T7 Ribomax kit (Promega). Injections and soaking were then performed as described [63,64]. Worms were analyzed 18–24 hours after injection except for *cdc-42(RNAi) *and *arx-1(RNAi) *embryos, which were imaged 30–36 hours after injection. We controlled for the efficacy and variability of RNAi by scoring for lethality and comparing phenotypes manifested in our treated embryos with published embryonic phenotypes of strong genetic alleles, where such information was available.

For phenotypic series of *actin(RNAi)*, each batch of dsRNA was calibrated for reproducibility of phenotypes and the same batch of dsRNA was used to quantify the amount of actin protein in the gonad over time. For antibody staining, groups of 7–10 worms injected within 15 minute intervals were fixed and stained at 6 h, 7 h, 8 h, 9 h, 10 h, 11 h, or 12 h after injection, and fluorescence intensity measured by Openlab 3.1.7 software. Series of timed gonad fixations were repeated 3 times for each batch of dsRNA, with similar trends in the levels of actin protein detected over time (Figure 1A and 1B show data from one representative series). Class I phenotypes were observed as early as 6–8 hours after injection depending on the batch of dsRNA used. Subsequent classes occurred as follows: Class II (1 hour after Class I), Class III (1.5–2 hours after Class I) and Class IV (2–3 hours after Class I). We assume that the variability in timing was due to variations in concentration of dsRNA in different preparations (which were not quantified).

### Live imaging and analysis of GFP fluorescence

For embryo observation, gravid worms were isolated in egg buffer and dissected on coverslips, which were then inverted in 2% or 4% agarose pads that were sealed with Vaseline. Observation and recordings for DIC and fixed fluorescent images were performed using a Leica DMLA microscope using a 100× (1.3 NA) objective with either an ORCA-ER (Hammamatsu C4742–95) digital camera. Fluorescent confocal images were obtained using the Spinning Disk Confocal system (Improvision) with an Electron Multiplier CCD camera (Hammamatsu C9100), a Ludl motorized XY stage, and an Improvision Piezo Focus Drive. The GFP::MOE strain was imaged at the cortex with a 63× (1.46 NA) objective by taking a 7-step (1 μm) Z-stack at the cortex every 10 s, at 75% laser power, 80 ms exposure, and a sensitivity of 169. Images were processed to a 3D-max projection using Volocity software and Adobe Photoshop 7.0 (Adobe Systems). Actin comet speed, length, and number were determined by taking an 8-step (1 μm) Z-stack through the top half of the embryo using the CARV confocal imager (BD Biosciences) or the Spinning Disk Confocal system (Improvision) and the Electron Multiplier CCD camera (Hamamatsu C9100); the acquisition system was controlled by OpenLab 3.1.7 or Volocity 4.1 (Improvision). Comets that stayed in one focal plane for 3 or more frames were used for measurement of speed and length. To quantify the number of comets present during different stages of the cell cycle, a 7 μm Z-stack with a 1 μm step was taken and the number of observed comets was recorded during five intervals of 3–4 minutes each between the following six timepoints: ~3.5 minutes before pseudocleavage, pseudocleavage, prometaphase, metaphase, completion of cytokinesis, and ~3.5 minutes after cytokinesis.

### Immunocytochemistry

For fixation and visualization of the F-actin cytoskeleton in the early embryo we used the protocol described in [23]. Gonad dissections, fixation and immunohistochemistry were carried out as described [65] for 6 timepoints after injection with *act-1 *dsRNA. Antibodies and dilutions were as follows: 1:1000 rabbit anti-GFP primary antibody (Abcam) or 1:200 monoclonal mouse anti-Actin Clone C4 (MP Biomedicals) with 1:1000 fluorescein isothiocyanate (FITC)-conjugated secondary anti-rabbit or anti-mouse IgG antibodies (Jackson Immuno Research). Rhodamine-conjugated phalloidin (1 unit/200 μl; Molecular Probes) was included with the secondary antibody to label F-actin specifically. Slides were washed in PBS and mounted in Vectashield (Vector Labs Inc. #H-1000).

## Authors' contributions

NV carried out all experiments and drafted the manuscript, FP conceived of the study, and FP, NV and KCG participated in the design and coordination of experiments and writing the manuscript. All authors read and approved the final manuscript.

## Supplementary Material

Additional File 1 **Supplementary Figure 1 **– ClustalW multiple DNA sequence alignment of conceptually spliced coding sequences for the three embryonic actins with the PCR product (sjj_T04C12.6) used as the template for *act-1(RNAi) *dsRNA. The top chart shows that all three actins (labeled "act-1", "act-2", and "act-3") show a high percent identity to each other and to the PCR product used for RNAi (labeled "act_1dsRNA"). Very high similarity extends across an extensive portion of all three actin sequences, including several long stretches of 100% identity, indicating that all three embryonic actins are depleted by *act-1(RNAi) *in this study. Click here for file

Additional File 4 **Movie 1 **– Embryogenesis in wild type (DIC and GFP::HIS) Click here for file

Additional File 5 **Movie 2 **– Class I *actin(RNAi) *(DIC) Click here for file

Additional File 6 **Movie 3 **– Class II *actin(RNAi) *(DIC) Click here for file

Additional File 7 **Movie 4 **– Class III *actin(RNAi) *(DIC) Click here for file

Additional File 8 **Movie 5 **– Class IV *actin(RNAi) *(DIC) Click here for file

Additional File 2 **Supplementary Figure 2 **– *actin(RNAi) *affects egg production and fertilization. Young adult hermaphrodites, either untreated [N = 3 for the N2 (WT) strain] or injected with *act-1 *dsRNA [N = 7 for N2; N = 13 for the PF100 (GFP::MOE) strain], were individually transferred to a new plate every 8 hrs. Plates were then scored for fertilized eggs (characterized by the presence of an eggshell and oval shape) and unfertilized oocytes (characterized by a large endomitotic nucleus and no eggshell). The proportion of unfertilized oocytes and fertilized eggs (Y-axis) was then plotted against the six successive time intervals (X-axis) for each strain and treatment scored (total number of eggs and oocytes scored are given in parentheses). The graph shows that after dsRNA injection, an increasing proportion of oocytes laid over time are unfertilized, and egg production eventually ceases altogether by 48 hrs, whereas WT animals continue to lay fertilized eggs at approximately the same rate. Click here for file

Additional File 3 **Supplementary Table 1 – Summary of phenotypic defects in *actin(RNAi) *embryos**. The range of normal and abnormal events observed and their occurrence in the four phenotypic classes of actin-depleted embryos vs. WT embryos is shown. Symbols: (+), observed; (-), not observed; n/a, not applicable. Click here for file

Additional File 9 **Movie 6 **– Early embryogenesis in GFP::MOE strain Click here for file

Additional File 10 **Movie 7 **– *par-6(RNAi) *of GFP::MOE strain Click here for file

Additional File 11 **Movie 8 **– *par-2(RNAi) *of GFP::MOE strain Click here for file

Additional File 12 **Movie 9 **– *par-1(RNAi) *of GFP::MOE strain Click here for file

Additional File 13 **Movie 10 **– *cdc-42(RNAi) *of GFP::MOE strain Click here for file

Additional File 14 **Movie 11 **– *pfn-1(RNAi) *of GFP::MOE strain Click here for file

Additional File 15 **Movie 12 **– *nmy-2(RNAi) *of GFP::MOE strain Click here for file

Additional File 16 **Movie 13 **– *arx-1(RNAi) *of GFP::MOE strain Click here for file

Additional File 17 **Movie 14 **– Actin comets in GFP::MOE strain Click here for file
