# Supplementary material for: Diverse roles of actin in C. elegans early embryogenesis
Source: BMC Dev Biol. 2007 Dec 24;7:142. doi: 10.1186/1471-213X-7-142 (PMC2323969; doi:10.1186/1471-213X-7-142)
Supplement: Additional File 1 — Supplementary Figure 1 – ClustalW multiple DNA sequence alignment of conceptually spliced coding sequences for the three embryonic actins with the PCR product (sjj_T04C12.6) used as the template for act-1(RNAi) dsRNA. The top chart shows that all three actins (labeled "act-1", "act-2", and "act-3") show a high percent identity to each other and to the PCR product used for RNAi (labeled "act_1dsRNA"). Very high similarity extends across an extensive portion of all three actin sequences, including several long stretches of 100% identity, indicating that all three embryonic actins are depleted by act-1(RNAi) in this study. [file 1471-213X-7-142-S1.DOC]

SeqA Name Len(nt) SeqB Name Len(nt) Score

===========================================================

1 act_1dsRNA 1199 2 act_1 1459 97

1 act_1dsRNA 1199 3 act_2 1418 90

1 act_1dsRNA 1199 4 act_3 2809 98

2 act_1 1459 3 act_2 1418 83

2 act_1 1459 4 act_3 2809 86

3 act_2 1418 4 act_3 2809 83

===========================================================

act_1dsRNA ----------------------------------------------TCCCCACGTGT--- 11

act_1 ------------------------------------------------------------

act_2 ------------------------------------------------------------

act_3 TTCGTGTTCTTTTCCAATTTCTTTTTCTTTCATAAGCTCCTTTTATATCTTAAATCTAAA 1200

act_1dsRNA TCTGTGTTATATTATCAATTTAATTT---TTCAGGTACATTAAAAACTAATCAAA-ATGT 67

act_1 ------------------TTTAATTT---TTCAGGTACATTAAAAACTAATCAAA-ATGT 38

act_2 -----------------------------GCTAAGTTCCTCCTAATCTAATAAATCATGT 31

act_3 CCTTTCTCATTTTTAAAATATAACTTATTTTCAGGTACATTAAAAACTAATCAGA-ATGT 1259

* ** * * ** ***** * ****

act_1dsRNA GTGACGACGAGGTTGCCGCTCTTGTTGTAGACAATGGATCCGGAATGTGCAAGGCCGGAT 127

act_1 GTGACGACGAGGTTGCCGCTCTTGTTGTAGACAATGGATCCGGAATGTGCAAGGCCGGAT 98

act_2 GTGACGACGATGTTGCCGCTCTCGTAGTTGACAATGGATCCGGAATGTGCAAAGCTGGAT 91

act_3 GTGACGACGAGGTTGCCGCTCTTGTTGTAGACAATGGATCCGGAATGTGCAAGGCCGGAT 1319

********** *********** ** ** *********************** ** ****

act_1dsRNA TCGCCGGAGACGACGCTCCACGCGCCGTGTTCCCATCCATTGTCGGAAGACCACGTCATC 187

act_1 TCGCCGGAGACGACGCTCCACGCGCCGTGTTCCCATCCATTGTCGGAAGACCACGTCATC 158

act_2 TCGCTGGAGACGACGCTCCACGCGCCGTGTTCCCATCCATTGTCGGAAGACCTCGTCATC 151

act_3 TCGCCGGAGACGACGCTCCACGCGCCGTGTTCCCATCCATTGTCGGAAGACCACGTCATC 1379

**** *********************************************** *******

act_1dsRNA AAGGAGTCATGGTCGGTATGGGACAGAAGGACTCGTACGTCGGAGACGAGGCCCAATCCA 247

act_1 AAGGAGTCATGGTCGGTATGGGACAGAAGGACTCGTACGTCGGAGACGAGGCCCAATCCA 218

act_2 AAGGAGTCATGGTCGGTATGGGACAGAAAGACTCGTACGTCGGAGACGAGGCCCAATCCA 211

act_3 AAGGAGTCATGGTCGGTATGGGACAGAAGGACTCGTACGTCGGAGACGAGGCCCAATCCA 1439

**************************** *******************************

act_1dsRNA AGAGAGGTAAATAATTAATACATTCGAT-GATTAAATTTATGCGTACTATTTCAGGTATC 306

act_1 AGAGAGGTAAATAATTAATACATTCGAT-GATTAAATTTATGCGTACTATTTCAGGTATC 277

act_2 AGAGAGGTAAATTTTCAAAAAATTTGACCGATTGGAAATAGTTGT----TTTTAGGTATC 267

act_3 AGAGAGGTAAATAATTAATACATTCGAT-GATTAAATTTATGCGTACTATTTCAGGTATC 1498

************ * ** * *** ** **** * ** ** *** *******

act_1dsRNA CTTACCCTCAAGTACCCAATTGAGCACGGTATCGTCACCAACTGGGATGATATGGAGAAG 366

act_1 CTTACCCTCAAGTACCCAATTGAGCACGGTATCGTCACCAACTGGGATGATATGGAGAAG 337

act_2 CTTACCCTTAAGTACCCAATTGAGCATGGTATCGTTACCAACTGGGACGACATGGAAAAA 327

act_3 CTTACCCTCAAGTACCCAATTGAGCACGGTATCGTCACCAACTGGGATGATATGGAGAAG 1558

******** ***************** ******** *********** ** ***** **

act_1dsRNA ATCTGGCATCACACCTTCTACAATGAGCTTCGTGTTGCCCCAGAAGAGCACCCAGTCCTC 426

act_1 ATCTGGCATCACACCTTCTACAATGAGCTTCGTGTTGCCCCAGAAGAGCACCCAGTCCTC 397

act_2 ATCTGGCATCACACTTTCTACAACGAGCTTCGCGTTGCCCCAGAAGAGCACCCAGTACTT 387

act_3 ATCTGGCATCACACCTTCTACAATGAGCTTCGTGTTGCCCCAGAAGAGCACCCAGTCCTC 1618

************** ******** ******** *********************** **

act_1dsRNA CTCACTGAAGCCCCACTCAATCCAAAGGCTAACCGTGAAAAGATGACCCAAATCATGTTC 486

act_1 CTCACTGAAGCCCCACTCAATCCAAAGGCTAACCGTGAAAAGATGACCCAAATCATGTTC 457

act_2 CTCACTGAAGCCCCACTCAATCCAAAGGCTAACCGTGAAAAGATGACCCAAATCATGTTC 447

act_3 CTCACTGAAGCCCCACTCAATCCAAAGGCTAACCGTGAAAAGATGACCCAAATCATGTTC 1678

************************************************************

act_1dsRNA GAGACCTTCAACACCCCAGCCATGTATGTCGCCATCCAAGCTGTCCTCTCCCTCTACGCT 546

act_1 GAGACCTTCAACACCCCAGCCATGTATGTCGCCATCCAAGCTGTCCTCTCCCTCTACGCT 517

act_2 GAGACCTTCAATACCCCAGCCATGTATGTCGCCATCCAAGCTGTCCTCTCCCTCTACGCT 507

act_3 GAGACCTTCAACACCCCAGCCATGTATGTCGCCATCCAAGCTGTCCTCTCCCTCTACGCT 1738

*********** ************************************************

act_1dsRNA TCCGGACGTACCACCGGAGTCGTCCTCGACTCTGGAGATGGTGTCACCCACACCGTCCCA 606

act_1 TCCGGACGTACCACCGGAGTCGTCCTCGACTCTGGAGATGGTGTCACCCACACCGTCCCA 577

act_2 TCCGGACGTACCACCGGAATCGTCCTCGACTCTGGAGATGGTGTTACCCACACAGTCCCA 567

act_3 TCCGGACGTACCACCGGAGTCGTCCTCGACTCTGGAGATGGTGTCACCCACACCGTCCCA 1798

****************** ************************* ******** ******

act_1dsRNA ATCTACGAAGGATATGCCCTCCCACACGCCATCCTCCGTCTTGACTTGGCTGGACGTGAT 666

act_1 ATCTACGAAGGATATGCCCTCCCACACGCCATCCTCCGTCTTGACTTGGCTGGACGTGAT 637

act_2 ATCTACGAAGGATATGCCCTCCCACACGCCATCCTCCGTCTTGACTTGGCTGGACGTGAT 627

act_3 ATCTACGAAGGATATGCCCTCCCACACGCCATCCTCCGTCTTGACTTGGCTGGACGTGAT 1858

************************************************************

act_1dsRNA CTTACTGATTACCTCATGAAGATCCTTACCGAGCGTGGTTACTCTTTCACCACCACCGCT 726

act_1 CTTACTGATTACCTCATGAAGATCCTTACCGAGCGTGGTTACTCTTTCACCACCACCGCT 697

act_2 CTTACTGATTACCTCATGAAGATCCTTACCGAGCGTGGTTACTCTTTCACCACCACCGCT 687

act_3 CTTACTGATTACCTCATGAAGATCCTTACCGAGCGTGGTTACTCTTTCACCACCACCGCT 1918

************************************************************

act_1dsRNA GAGCGTGAAATCGTCCGTGACATCAAGGAGAAGCTCTGCTACGTCGCCCTCGACTTCGAG 786

act_1 GAGCGTGAAATCGTCCGTGACATCAAGGAGAAGCTCTGCTACGTCGCCCTCGACTTCGAG 757

act_2 GAGCGTGAAATCGTCCGTGACATCAAGGAGAAGCTTTGTTACGTCGCCCTCGATTTCGAG 747

act_3 GAGCGTGAAATCGTCCGTGACATCAAGGAGAAGCTCTGCTACGTCGCCCTCGACTTCGAG 1978

*********************************** ** ************** ******

act_1dsRNA CAAGAAATGGCCACCGCCGCTTCTTCCTCTTCCCTCGAGAAGTCCTACGAACTTCCTGAC 846

act_1 CAAGAAATGGCCACCGCCGCTTCTTCCTCTTCCCTCGAGAAGTCCTACGAACTTCCTGAC 817

act_2 CAAGAAATGGCCACCGCCGCTTCTTCCTCTTCCCTCGAGAAGTCCTACGAACTTCCTGAC 807

act_3 CAAGAAATGGCCACCGCCGCTTCTTCCTCTTCCCTCGAGAAGTCCTACGAACTTCCTGAC 2038

************************************************************

act_1dsRNA GGACAAGTCATCACCGTCGGAAACGAACGTTTCCGTTGCCCAGAGGCTATGTTCCAGCCA 906

act_1 GGACAAGTCATCACCGTCGGAAACGAACGTTTCCGTTGCCCAGAGGCTATGTTCCAGCCA 877

act_2 GGACAAGTCATCACTGTTGGAAACGAACGCTTCCGTTGCCCAGAGGCTCTGTTCCAACCA 867

act_3 GGACAAGTCATCACCGTCGGAAACGAACGTTTCCGTTGCCCAGAGGCTATGTTCCAGCCA 2098

************** ** *********** ****************** ******* ***

act_1dsRNA TCCTTCTTGGGTATGGAGTCCGCCGGAATCCACGAGACTTCTTACAACTCCATCATGAAG 966

act_1 TCCTTCTTGGGTATGGAGTCCGCCGGAATCCACGAGACTTCTTACAACTCCATCATGAAG 937

act_2 TCCTTCTTGGGTATGGAATCTGCAGGAATCCACGAGACTTCTTACAACTCCATCATGAAG 927

act_3 TCCTTCTTGGGTATGGAGTCCGCCGGAATCCACGAGACTTCTTACAACTCCATCATGAAG 2158

***************** ** ** ************************************

act_1dsRNA TGCGACATTGATATCCGTAAGGACTTGTACGCCAACACTGTTCTTTCCGGAGGAACCACC 1026

act_1 TGCGACATTGATATCCGTAAGGACTTGTACGCCAACACTGTTCTTTCCGGAGGAACCACC 997

act_2 TGCGACATTGATATCCGTAAGGACTTGTACGCCAACACTGTTCTTTCCGGAGGAACCACC 987

act_3 TGCGACATTGATATCCGTAAGGACTTGTACGCCAACACTGTTCTTTCCGGAGGAACCACC 2218

************************************************************

act_1dsRNA ATGTACCCAGGAATTGCTGATCGTATGCAGAAGGAAATCACCGCTCTTGCCCCATCGTAA 1086

act_1 ATGTACCCAGGAATTGCTGATCGTATGCAGAAGGAAATCACCGCTCTTGCCCCATCGTAA 1057

act_2 ATGTACCCAGGAATTGCTGATCGTATGCAGAAGGAAATCACCGCTCTTGCCCCATCGTAA 1047

act_3 ATGTACCCAGGAATTGCTGATCGTATGCAGAAGGAAATCACCGCTCTTGCCCCATCGTAA 2278

************************************************************

act_1dsRNA GTTTTCTATTTTTGTTTCAGTTAACAAATTAAATTGATTTTTTTT-CAGAACCATGAAGA 1145

act_1 GTTTTCTATTTTTGTTTCAGTTAACAAATTAAATTGATTTTTTTT-CAGAACCATGAAGA 1116

act_2 GTTTTCTATTTTTGTTTTAGTTAACAAATTAATTTGATTTTTTTT-CAGAACCATGAAGA 1106

act_3 GTTTTCTATTTTTGTTTCAGTTAACAAATTAATTTGATTTTTTTTTCAGAACCATGAAGA 2338

***************** ************** ************ **************

act_1dsRNA TCAAGATCATCGCCCCACCAGAGCGCAAGTACTCCGTCTGGATCGGAGGATCTA------ 1199

act_1 TCAAGATCATCGCCCCACCAGAGCGCAAGTACTCCGTCTGGATCGGAGGATCTATCCTCG 1176

act_2 TCAAGATCATCGCCCCACCAGAGCGCAAGTACTCCGTCTGGATCGGAGGATCTATCCTCG 1166

act_3 TCAAGATCATCGCCCCACCAGAGCGCAAGTACTCCGTCTGGATCGGAGGATCTATCCTCG 2398

******************************************************
