# Supplementary figures and images for: Diverse roles of actin in C. elegans early embryogenesis
Source: BMC Dev Biol. 2007 Dec 24;7:142. doi: 10.1186/1471-213X-7-142 (PMC2323969; doi:10.1186/1471-213X-7-142)

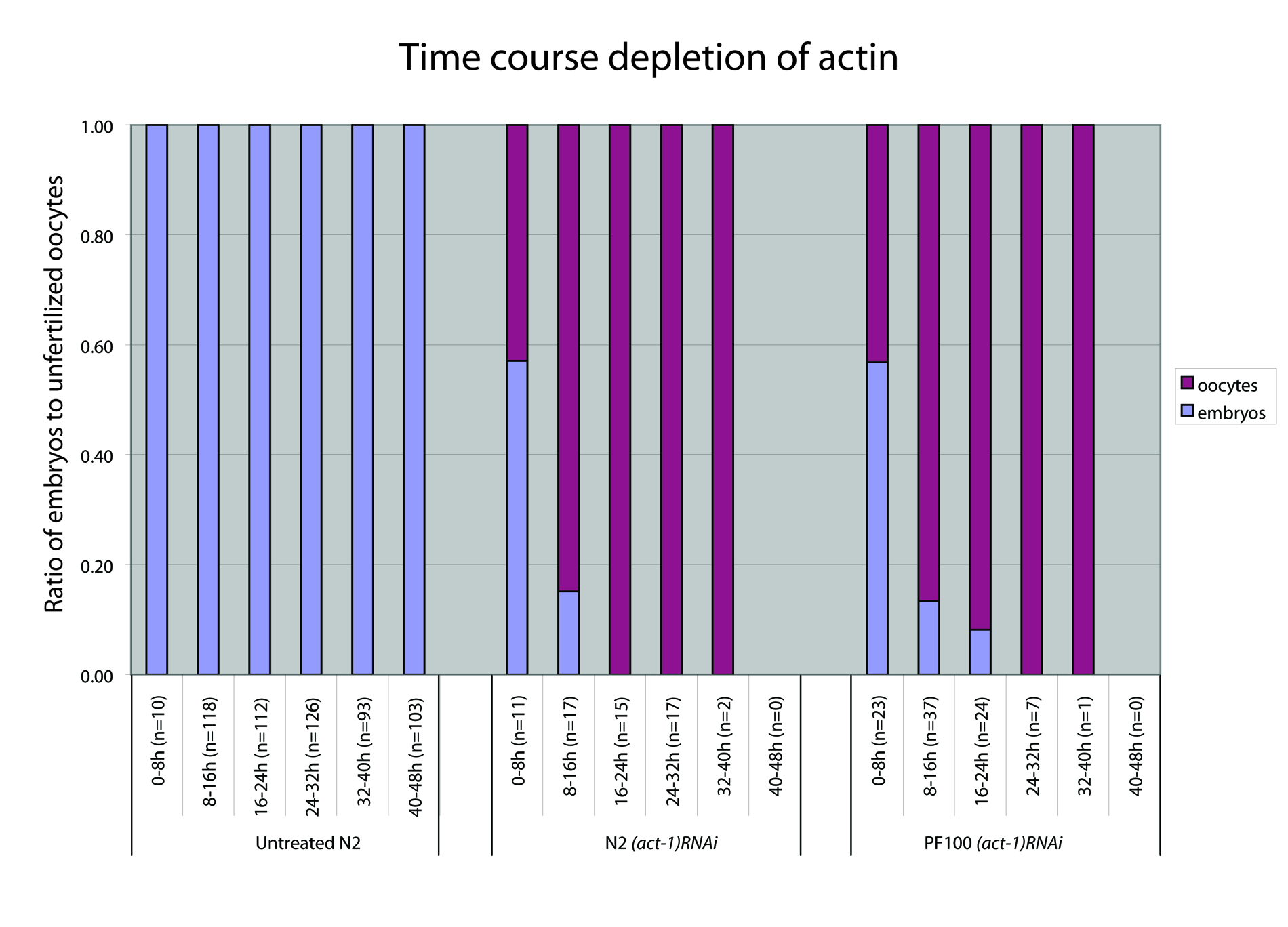

Supplement: Additional File 2 — Supplementary Figure 2 – actin(RNAi) affects egg production and fertilization. Young adult hermaphrodites, either untreated [N = 3 for the N2 (WT) strain] or injected with act-1 dsRNA [N = 7 for N2; N = 13 for the PF100 (GFP::MOE) strain], were individually transferred to a new plate every 8 hrs. Plates were then scored for fertilized eggs (characterized by the presence of an eggshell and oval shape) and unfertilized oocytes (characterized by a large endomitotic nucleus and no eggshell). The proportion of unfertilized oocytes and fertilized eggs (Y-axis) was then plotted against the six successive time intervals (X-axis) for each strain and treatment scored (total number of eggs and oocytes scored are given in parentheses). The graph shows that after dsRNA injection, an increasing proportion of oocytes laid over time are unfertilized, and egg production eventually ceases altogether by 48 hrs, whereas WT animals continue to lay fertilized eggs at approximately the same rate. [file 1471-213X-7-142-S2.TIFF]
