# Supplementary material for: Diverse roles of actin in C. elegans early embryogenesis
Source: BMC Dev Biol. 2007 Dec 24;7:142. doi: 10.1186/1471-213X-7-142 (PMC2323969; doi:10.1186/1471-213X-7-142)
Supplement: Additional File 3 — Supplementary Table 1 – Summary of phenotypic defects in actin(RNAi) embryos. The range of normal and abnormal events observed and their occurrence in the four phenotypic classes of actin-depleted embryos vs. WT embryos is shown. Symbols: (+), observed; (-), not observed; n/a, not applicable. [file 1471-213X-7-142-S3.DOC]

| **Phenotype** | **Wild Type** | **Class I** | **Class II** | **Class III** | **Class IV** |
| --- | --- | --- | --- | --- | --- |
| Cortical Ruffling | + | - | - | - | - |
| Pseudocleavage | + | - | - | - | - |
| Polar body extrusion | + | + | + | + | - |
| Polar body re-absorption | - | - | - | + | n/a |
| Paternal pronucleus associates closely with cortex | + | + | - | - | - |
| Pronuclei meet centrally | - | - | + | + | + |
| Decondensed chromosomes | - | - | - | + | + |
| Chromosome bridges during anaphase | - | - | - | + | + |
| Reduced elongation of the central spindle | - | - | - | + | + |
| Contractile ring forms | + | + | + | + | - |
| Asymmetric first cell division | + | + | - | n/a | n/a |
| Asynchronous second cell division | + | - | - | n/a | n/a |
| AB spindle perpendicular to P1 spindle | + | + | - | n/a | n/a |
| Disorganized furrows | - | - | - | + | n/a |
